# Cooking breakfast after a brain injury

**DOI:** 10.3389/fnbeh.2014.00272

**Published:** 2014-09-02

**Authors:** Annick N. Tanguay, Patrick S. R. Davidson, Karla V. Guerrero Nuñez, Mark B. Ferland

**Affiliations:** ^1^School of Psychology, University of OttawaOttawa, ON, Canada; ^2^Bruyère Research Institute, University of OttawaOttawa, ON, Canada; ^3^Canadian Partnership for Stroke Recovery, Heart and Stroke Foundation of CanadaOttawa, ON, Canada; ^4^The Robin Easey Centre, Ottawa Hospital Rehabilitation CentreOttawa, ON, Canada; ^5^Ottawa Hospital Research InstituteOttawa, ON, Canada

**Keywords:** cooking, acquired brain injury, independent activities of daily living, executive functions, simulated/computerized cooking, ecological validity, rehabilitation

## Abstract

Acquired brain injury (ABI) often compromises the ability to carry out instrumental activities of daily living such as cooking. ABI patients' difficulties with executive functions and memory result in less independent and efficient meal preparation. Accurately assessing safety and proficiency in cooking is essential for successful community reintegration following ABI, but *in vivo* assessment of cooking by clinicians is time-consuming, costly, and difficult to standardize. Accordingly, we examined the usefulness of a computerized meal preparation task (the Breakfast Task; Craik and Bialystok, 2006) as an indicator of real life meal preparation skills. Twenty-two ABI patients and 22 age-matched controls completed the Breakfast Task. Patients also completed the Rehabilitation Activities of Daily Living Survey (RADLS; Salmon, 2003) and prepared actual meals that were rated by members of the clinical team. As expected, the ABI patients had significant difficulty on all aspects of the Breakfast Task (failing to have all their foods ready at the same time, over- and under-cooking foods, setting fewer places at the table, and so on) relative to controls. Surprisingly, however, patients' Breakfast Task performance was not correlated with their *in vivo* meal preparation. These results indicate caution when endeavoring to replace traditional evaluation methods with computerized tasks for the sake of expediency.

## Introduction

The executive functions are a family of processes that support goal-setting, planning, organizing, monitoring, and the flexible control of cognition and behavior. Although executive dysfunction is one of the most common and clinically significant consequences of brain injury, there remains much controversy on exactly how to assess it (Spooner and Pachana, 2006; Lowenstein and Acevedo, 2010). For decades, the dominant strategy has been to employ a handful of brief, non-natural tasks, for example, the Wisconsin Card Sorting Test (WCST). This approach has many advantages: Tests can be standardized in their administration, scoring, and interpretation; can (or, at least, can strive to) isolate one or more putative executive processes from others (e.g., Stuss et al., 2000; Barceló and Knight, 2002; Specht et al., 2009); and can be based on increasingly sophisticated neurocognitive models, allowing for patient data to be compared against human neuroimaging and animal physiological and lesion findings (e.g., Nyhus and Barceló, 2009). This approach is not without its difficulties, however, including the often surprisingly poor generalizability to behavior in the real world: Low scores on classical measures of executive function such as the WCST do not necessarily imply poor executive behavior in everyday life, and, conversely, good performance on classical executive measures can be accompanied by severely dysexecutive comportment in everyday life (e.g., Eslinger and Damasio, 1985; Chevignard et al., 2000; Andrés and Van der Linden, 2002; Fortin et al., 2003; Barker et al., 2004; Manchester et al., 2004).

Recently, an alternative approach has taken root. It entails the use of more complex tasks that incorporate multiple executive functions to carry out a scenario from the real world, such as running errands (Shallice and Burgess, 1991; Knight et al., 2002) or managing the front desk of a hotel (Manly et al., 2002; see also Lamberts et al., 2010; for a review see Poulin et al., 2013). The goal of using these more representative (i.e., corresponding more closely in form and context to situations outside the clinic/lab) scenarios is to yield results that are more generalizable (i.e., enabling better prediction of performance outside the clinic/lab; Burgess et al., 2006) than classical measures of executive function such as the WCST (Chaytor and Schmitter-Edgecombe, 2003). Our goal here was to examine brain-injured patients' performance on one such scenario: Cooking a meal.

Cooking is a good example of a real world task that often draws heavily on executive functioning. The classic illustration of this comes from neurosurgeon Wilder Penfield (Penfield and Evans, 1935), who performed an extensive right frontal lobe resection on his sister. Her inability to orchestrate a small dinner a year later was seen as emblematic of her general problems with executive functioning: “She had planned to get a simple supper for one guest and four members of her own family…When the appointed hour arrived the food was all there, one or two things [were] on the stove, but the salad was not ready, the meat had not been started and she was distressed and confused by her long continued effort alone.” Myriad subsequent research has demonstrated that brain injuries impair cooking (Dawson and Chipman, 1995; Chevignard et al., 2000, 2008; Fortin et al., 2003; Corrigan et al., 2004; Godbout et al., 2005; Baguena et al., 2006; Lillie et al., 2010; Frisch et al., 2012) but cooking is not convincingly correlated with performance on traditional tests of executive functions (Semkovska et al., 2004; Baum et al., 2008; Chevignard et al., 2008, 2010; Yantz et al., 2010; Provencher et al., 2012).

**Figure 1:**
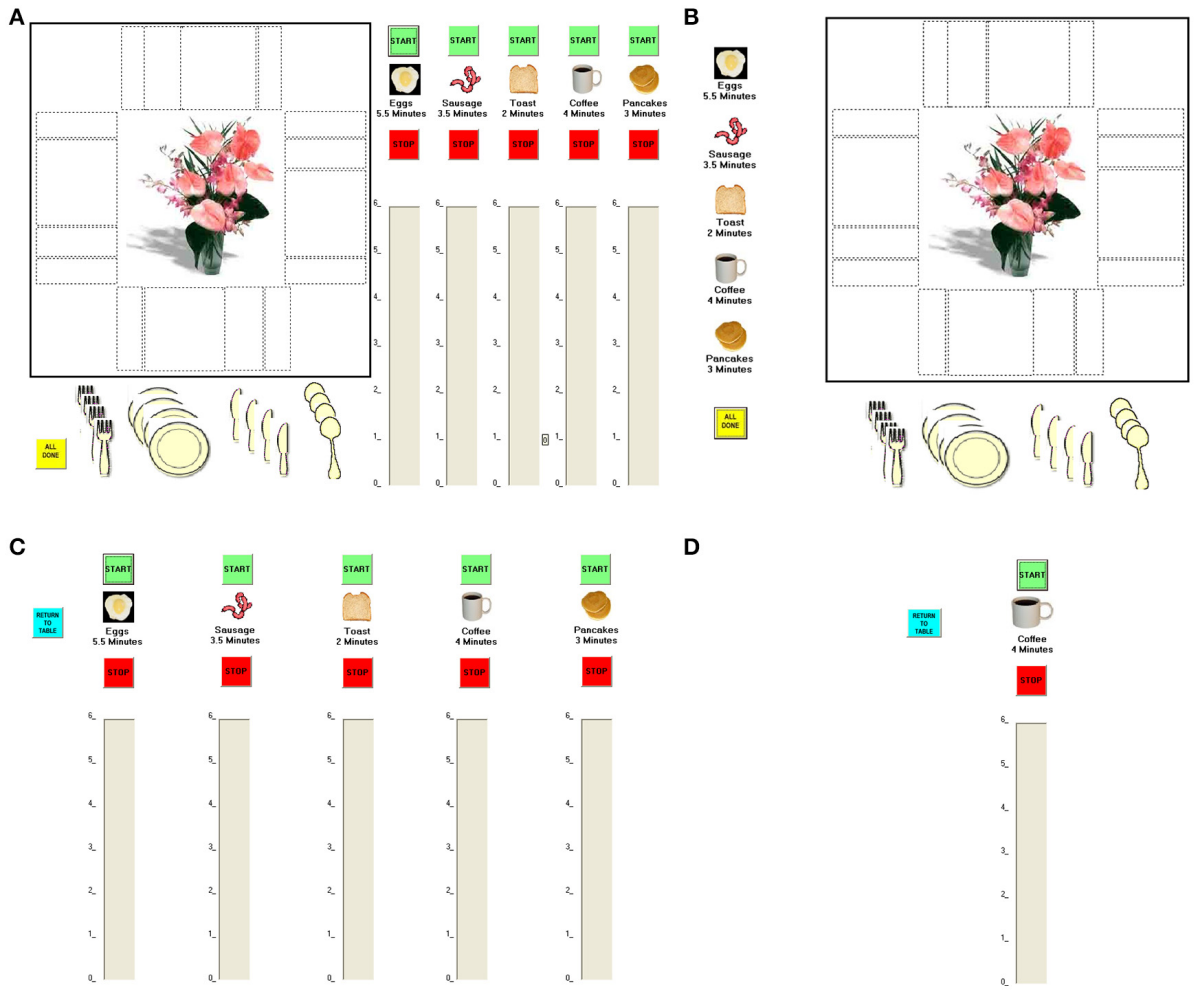
**The Breakfast Task versions. (A)** The 1-screen version: The five foods and the table are shown on a single screen. **(B,C)** The 2-screen version: The five foods are shown on one screen and the table on a separate one. **(B,D)** The 6-screen version: The five foods and the table are shown on a separate screen.

To strike a balance between the advantages of standardization and experimental control inherent to traditional tests of executive functions and the intricate and varying demands placed on executive functions by real-world scenarios, Craik and Bialystok (2006) developed the Breakfast Task (from Kerr, 1991). It is a computerized simulation in which participants use a touch screen to virtually “cook” five foods (each requiring a different cooking time) and ensure that they are all ready at the same time, while simultaneously setting the places at a virtual table. The task has three levels of difficulty, each thought to place heavier demands on executive functioning than the previous level (a *1-screen version*, in which all five foods and the table are shown on the same screen; a *2-screen version*, in which the five foods are shown on one screen and the table on a separate one, requiring participants to switch between the two screens; and a *6-screen version*, in which each of the five foods and the table are shown on a separate screen, requiring participants to switch among the six screens; Figure 1). Successful performance on the Breakfast Task (especially on the 6-screen version) requires participants to plan, multitask, hold different elements of one's plan and one's activities in mind while operating, monitor performance, and at times inhibit one behavior and switch to another. These are the hallmarks of executive functioning.

Healthy older adults, who on average have mild difficulties with executive functioning, performed more poorly on most aspects of the Breakfast Task than did young people, especially on the most executively-demanding 6-screen version (Craik and Bialystok, 2006). The only published study of neurological patients on the Breakfast Task of which we are aware involved Parkinson's disease (Bialystok et al., 2008). Although typically Parkinson's patients are thought of as having mild-to-moderate difficulties with planning and executive control, they performed as well as or better than older controls on most Breakfast Task measures. Though this result might at first glance seem surprising, the patients' good performance on breakfast-making came at a cost: They by and large neglected to set places at the virtual table. The authors hypothesized that this may have been a compensatory strategy initiated by the patients: Because they quickly realized they would have difficulty doing everything asked of them, they deliberately neglected the secondary task (i.e., place-setting) to ensure good performance on the main task (i.e., breakfast-making).

What does the Breakfast Task tell us about cooking in everyday life? The fact that it is representative of cooking, relatively easy to comprehend, and reported by participants to be enjoyable-yet-challenging (Craik and Bialystok, 2006), bodes well for its generalizability (Chaytor and Schmitter-Edgecombe, 2003; Burgess et al., 2006; Chan et al., 2008). As additional support, Craik and Bialystok cited data linking Breakfast Task performance with strategy use among older adults during real life meal preparation (Edwards and Ryan, 2004). Yet, although the Breakfast Task looks promising, at the moment there are still too few neuropsychological studies to know exactly what to make of it. To this end, here we examined the potential generalizability of the Breakfast Task to real cooking in people with acquired brain injuries (ABI). Accurately assessing safety and proficiency in cooking is essential for successful community reintegration following ABI, but *in vivo* assessment of cooking by clinicians is time-consuming, costly, and difficult to standardize. Accordingly, we examined the usefulness of the Breakfast Task as an indicator of real life meal preparation skills:

- We expected that ABI patients would be significantly impaired on most aspects of the Breakfast Task compared to healthy controls (especially on the 6-screen version, which is the most executively-demanding).
- It was an open question whether the ABI patients would show a pattern similar to the Bialystok et al. (2008) Parkinson's patients, neglecting the table-setting element of the task in an attempt to preserve performance on breakfast-making.
- We expected that ABI patients' performance on the Breakfast Task would be positively correlated with their performance on preparation of a real meal (as rated by a neuropsychologist and a life skills counselor).

## Materials and methods

### Participants

Twenty-two people with ABI participated in the study [7 females; mean age = 45.55 years (*SD* = 12.44), mean education = 14.14 years (*SD* = 2.87)]. Three additional ABI patients started but then declined to complete the full Breakfast Task, and were therefore excluded from all analyses. All ABI participants were English-speaking, and were free of acute psychiatric symptoms, hemiparesis in the dominant hand, and visual field cut/hemi-neglect. All had received services within the last 36 months from the Robin Easey Centre, a Transitional Living Rehabilitation Program staffed with a full team of health care providers for adults having sustained an ABI. The Program focuses on reducing levels of disability via training and use of compensatory strategies. In general, of the clients obtaining services at the Centre, approximately 40% have sustained a traumatic brain injury, with the remainder having suffered acquired brain insults of varying etiologies (e.g., encephalopathy, ruptured aneurysm, tumor). The average duration of stay within the residential program is 161 days (*SD* = 87). Typically, clients are past the most acute stages of recovery (i.e., more than 6 months post-insult) by the time they are admitted into the residential program. Most clients have undergone a stay in an acute care facility followed by an admission to an in-hospital rehabilitation program before being referred to services at the Robin Easy Centre. Others are already several years post-insult when admitted, and seeking to acquire the skills needed for greater living independence.

Twenty-two healthy controls also participated (14 females; mean age = 39.86 years, *SD* = 17.96, mean education = 16.61 years, *SD* = 2.35). Four additional control participants were excluded: Two for Montreal Cognitive Assessment scores below the cut-off of 26/30 (Nasredinne et al., 2005), one for difficulties understanding and/or following the instructions and using the touch screen, and one (a younger woman, randomly selected from 4) to improve the match in age and sex ratio with the ABI group. The control and patients groups were similar in age *t*_(42)_ = 1.220, *p* = 0.229, although they differed in sex distribution *x*^2^(1) = 4.464, *p* = 0.035, ϕ = 0.319 and years of education *t*_(42)_ = −3.137, *p* = 0.003. [Note that these demographic differences appeared not to be the drivers of the very large differences in performance between groups on the Breakfast Task: When we re-ran our analyses using a smaller control group (*n* = 15) more closely matched to the patients, we found essentially the same results on the Breakfast Task (ANCOVAs using the larger control group were precluded because assumptions of homogeneity of regression were not met). We have included the entire control sample in the current version, to give a fuller picture of normal performance.]

### Materials and instrumentation

All participants were assessed on the Breakfast Task, and in addition the ABI patients were assessed on a self-report measure and an *in vivo* cooking task. The Research Ethics Boards of the Ottawa Hospital Research Institute and the University of Ottawa committee approved the study.

#### The Breakfast Task

The Craik and Bialystok (2006) and Bialystok et al. (2008) computerized meal simulation task was completed using a touch-screen monitor. The main objectives were to cook the five breakfast food items [in the following order: eggs (ideal cooking time = 5.5 min/330 s), coffee 4 min/240 s, sausage 3.5 min/210 s, pancakes 3 min/180 s, and toast 2 min/120 s] thoroughly and to have them ready at the same time, while simultaneously setting places at a virtual table.

To start cooking a food item, the participants pressed its associated “Start” icon. This highlighted the name of the food item in green and made a blue bar appear on a timer, a vertical column. This blue bar dropped toward the zero mark, reflecting the remaining time, in real time, before the food would be ready. When the bar reached the zero mark, the participants pressed the “Stop” icon to stop the food cooking. No further indications were provided once the blue bar had reached the zero mark; consequently, cooking the food items required constant monitoring. When the “Stop” icon was pressed, the name of the food item was highlighted in red. The “Start” and “Stop” buttons could only be pressed once, thus participants could not stop the cooking of a food item if it had been started early.

The secondary objective was to set as many table settings as possible, setting repeatedly a table arranged to accommodate 4 guests. The location of the plate and utensils followed typical etiquette.

In the practice trials, the participants cooked two breakfast food items, whereas in the test trials, the participants cooked five breakfast foods. For both the demonstration and test trials, the participants completed three versions of the task. These versions of the task differed in their number of screens: 1-screen, 2-screen (one table setting screen and one cooking screen), or 6-screen (one table setting screen and a screen for each food item). The level of difficulty across the Breakfast Task is postulated to increase because of the greater executive/working memory demands. Like Craik and Bialystok (2006) and Bialystok et al. (2008) we presented the practice and test trials in a fixed order going from the 1-screen to the 2-screen, and ending with the 6-screen.

#### Self-report measure: Rehabilitation Activities of Daily Living Survey (RADLS)

Within a few days of patients' discharge from the residential program, trained life skills counselors administered the Rehabilitation Activities of Daily Living Survey (RADLS; Salmon, 2003). The RADLS is a survey that measures patients' perceived cognitive, emotional and physical impairments. It assesses daily living tasks such as bathing, climbing stairs, relating to friends and family, banking, etc. Participants report their percent of limitation related to each activity before and after onset of the injury/illness. Responses were reversed and averaged to reflect abilities from 0% = full assistance/cannot do at all to 100% = fully independent. Items are divided into 10 composite categories, two of which are of particular interest: Meals, and Cognitive Activities (e.g., paying bills, running errands).

#### *In vivo* cooking assessment

During the first 4 weeks of stay within the treatment facility, participants underwent a comprehensive assessment. Actual meal preparations were observed and evaluated by an occupational therapist and/or a life skills counselor. Patients were asked to prepare one meal a week that would feed themselves and other clients at the Centre (i.e., 3–5 persons in all). Clients were allowed to choose their own menu, but sometimes suggestions were made and consideration was given to maintaining preparation time within 1–1.5 h. The staff kept the context stable from 1 week to the next, and free from interruptions by other residents. The evaluators typically limited their involvement to observations and ratings, except if an obvious safety risk arose.

On the basis of 4 meal preparations, the occupational therapist and life skills counselor summarized their impressions of a patient's meal preparation skills. Also noted was the spontaneous use of strategies by the client and recommendations were made about additional training in meal preparation and suitable compensatory strategies. Such strategies might include separating planning from execution, strategies to better organize space, use of timers and alarms, modifying and simplifying recipes, use of adapted tools for the kitchen, keeping track of the passage of time, limiting the level of multi-tasking and so forth.

Subsequently, clients continued to prepare a similar group meal once a week for the remainder of their stay. These meal preparations were also supervised and evaluated by the occupational therapist or a life skills counselor. The meal preparations were used to teach clients new compensatory strategies. A discharge report was also prepared, including information about meal preparation skills, and any gains made with the use of compensatory strategies.

The neuropsychologist and a senior life skills counselor reviewed the admission and discharge reports for sections in relation to meal preparation. The rating guidelines were developed by the neuropsychologist to examine three dimensions of cooking: efficiency of execution, successful use of client-generated or trained compensatory strategies, and overall independence with meal preparation. Clients were rated relative to the range of abilities demonstrated across clients seen within the Residential Program. Both the team neuropsychologist and one senior life skills counselor familiarized themselves with the guidelines and both practiced using the method before rating participants. Subsequently, they used a 4-point Likert scale to rate each subject along the 3 dimensions (i.e., not effective to very effective strategy use, very slow to mildly/not slow, not at all/virtually not at all independent to very independent with meal preparation). The two raters obtained a good agreement; the intra-class correlation reached 0.875 (with 95% confidence intervals from 0.658 to 0.952).

### Procedure

The intake coordinator reviewed the list of clients who had received services from the Robin Easey Centre in the last 36 months and who met the inclusionary criteria for participation. The intake coordinator contacted the patients to describe the study, and invited them to participate. Patients did not receive compensation.

The control participants were recruited in order to match the ABI patients as a group on age, sex, and education. One author (Karla V. Guerrero Nuñez) tested the patients and trained another author (Annick N. Tanguay), who recruited and tested the controls. Testing conditions were otherwise similar for both groups (identical procedure, instructions, reminders, etc.). Controls were recruited from the community and an undergraduate psychology students' pool, and received, respectively, $10 or partial course credit. Some controls were tested at home for their convenience (note that the Robin Easy Centre acts as a temporary home for ABI patients).

The first step of each testing session involved a thorough description of the study and informed consent. Participants were reminded that they could withdraw from the study at any point during the experiment and that their participation was voluntary. We then obtained demographic information and for the patients corroborated it with their health care records when necessary, with their agreement.

The experimenter gave verbal instructions to participants and demonstrated the Breakfast Task. When the patients felt comfortable with the instructions, they moved on to completing the demonstration trials during which they could continue to ask questions. Before beginning each trial, the experimenter briefly reminded the participants of the main goals (i.e., having all food cooked and ready at the same time) and the secondary objective (i.e., setting the table as often as possible) of the task as well as other key points, such as taking note of the different cooking times and that the “Start” and “Stop” icons could only be pressed once. The experimenter sat at the back of the room to observe and take notes of the participants' performance. During the test trials, the experimenter no longer provided clarifications. The same fix order was used for practice and test trials: 1-screen, 2-screen, and 6-screen. It took approximately 45 min to complete the testing session, from obtaining consent to completing all levels of the Breakfast Task.

### Statistical analyses

We tested for Group differences (ABI patients, controls) and within-subject differences across the 3 Breakfast Task Versions (1-screen, 2-screen, 6-screen) using 2 × 3 mixed ANOVAs, following up with *post-hoc t*-tests where necessary. Because the majority of the scores are based on reaction times (with inherently positively-skewed distributions and some outliers, especially among the patients), we show raw scores in the Figures but performed log^−10^ transformations of the data before conducting the ANOVAs and *post-hocs*. For ease of interpretation and to provide a better indication of the Breakfast Task's potential clinical usefulness, we used raw scores when examining the relationship (Spearman's rho) between the Breakfast Task and real world indices of cooking ability.

## Results

### Comparisons between ABI and controls

#### Total task time

**Figure 2:**
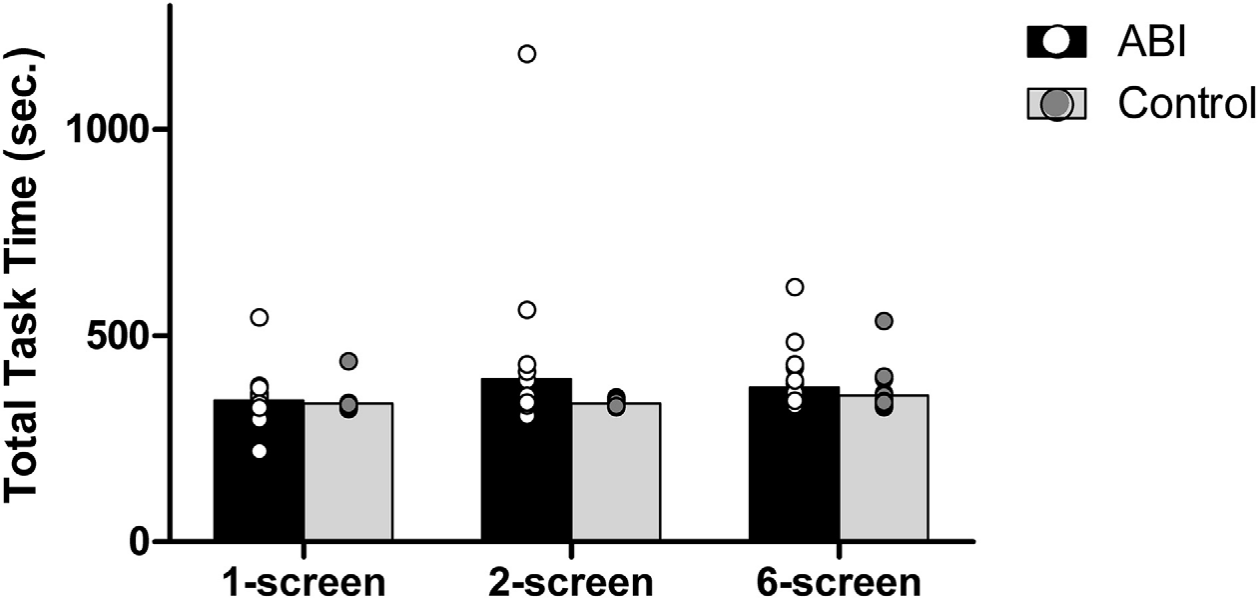
**Total task time in seconds**. Dots represent individual data points.

The Breakfast Task consists of 5 food items, with the eggs always taking the longest to cook (5.5 min = 330 s). Thus, on each version of the Breakfast Task the optimal total task time is 5.5 min. Taking less than 5.5 min would render the eggs under-cooked, whereas taking longer than 5.5 min would indicate a lack of efficiency/organization, with at least one breakfast item likely ending up cold or burned. Overall, the patients took only slightly longer than the controls to complete the task, *F*_(1, 42)_ = 2.161, *MSE* = 0.009, *p* = 0.149; η^2^ = 0.049 (see Supplementary Table 1 and Figure 2). The 3 versions of the Breakfast Task took different times to complete, *F*_(2, 84)_ = 4.595, MSE = 0.002, *p* = 0.013, η^2^ = 0.099. The 6-screen version (*M* = 2.559, *SD* = 0.058) took more time than the 1-screen version (*M* = 2.529, *SD* = 0.049), *t*_(43)_ = −3.165, *p* = 0.003. The 2-screen version (*M* = 2.55, *SD* = 0.091) did not differ significantly from either the 1-screen, *t*_(43)_ = −1.927, *p* = 0.061, or 6-screen version, *t*_(43)_ = −0.821, *p* = 0.416.

The interaction between Group and Version tended toward but did not obtain significance, *F*_(2, 84)_ = 2.277, MSE = 0.002, *p* = 0.109, η^2^ = 0.051.

#### Average discrepancy in cooking time

**Figure 3:**
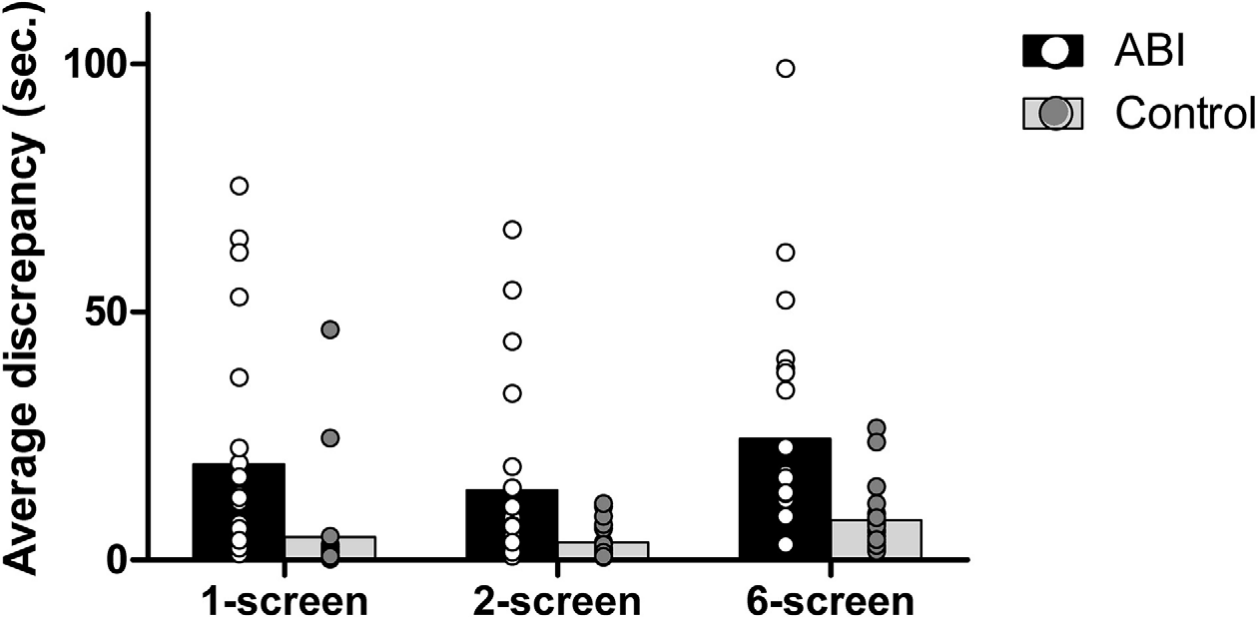
**Average discrepancy in seconds**.

As in real life, each of the Breakfast Task's foods has an ideal cooking time, which is computed and displayed for participants (e.g., the eggs take 5.5 *min* = 330 s). Any deviation from the ideal cooking time will lead to an over- or under-cooked item. We obtained the average discrepancy in cooking time by computing the difference between the actual cooking time of each food and its ideal cooking time and then averaging the absolute scores across each of the 5 foods. The ABI patients showed a greater discrepancy than controls, *F*_(1, 42)_ = 21.403, MSE = 0.295, *p* < 0.001, η^2^ = 0.338 (see Supplementary Table 1 and Figure 3). We also found a main effect of Breakfast Task Version, *F*_(2, 84)_ = 18.237, MSE = 0.082, *p* < 0.001, η^2^ = 0.303, but no interaction between Group and Version, *F*_(2, 84)_ = 1.230, MSE = 0.082, *p* = 0.297, η^2^ = 0.028. The 1-screen (*M* = 0.75, *SD* = 0.533) and 2-screen (*M* = 0.744, *SD* = 0.422) versions did not differ from one another, *t*_(43)_ = 0.094, *p* = 0.926, but both involved lower average discrepancy scores than the 6-screen version (*M* = 1.066, *SD* = 0.371), *t*_(43)_ ≤ −4.734, *p* < 0.001 and *t*_(43)_ = −5.991, *p* = 0.001, respectively.

#### Early stopping vs. late stopping

The “average discrepancy in cooking time” scores (reported immediately above) indicated that the ABI patients stopped cooking their foods at the wrong times. We then looked more closely at these data to find out whether people were stopping too soon or too late. To obtain the average discrepancy in cooking time, each of the food items' ideal cooking times had been subtracted from their actual cooking times. Negative discrepancies result from undercooking (i.e., early stopping) and positive discrepancies from overcooking (i.e., late stopping). Negative and positive discrepancies of the 5 food items were averaged separately to obtain a measure representing early and late stopping times, respectively.

**Figure 4:**
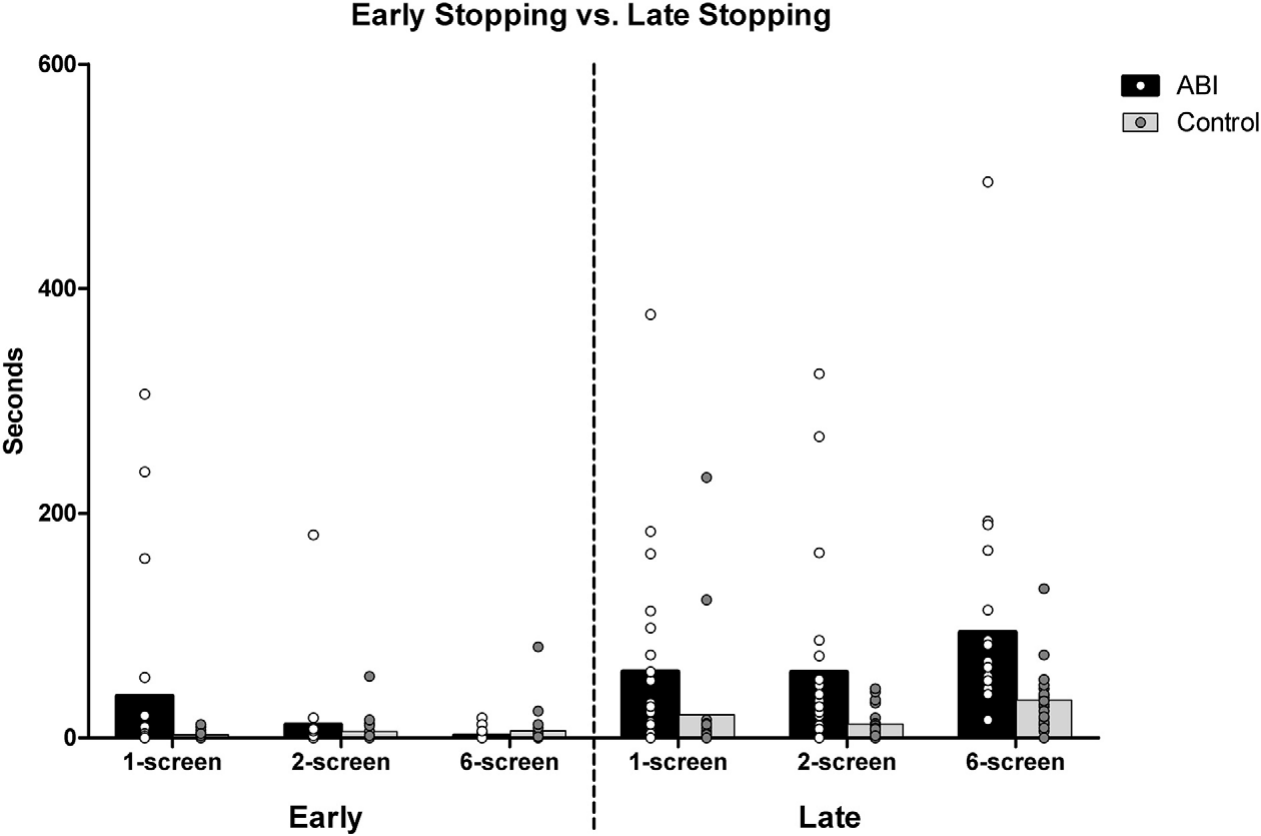
**Average early and late stopping discrepancies in seconds**.

When participants undercooked their foods (i.e., stopped their foods too soon), the two groups were not significantly different overall, *F*_(1, 42)_ = 0.956, MSE = 0.523, *p* = 0.334, η^2^ = 0.022 (see Supplementary Table 1 and Figure 4). There was a main effect of Breakfast Task version, *F*_(2, 84)_ = 4.574, MSE = 0.194, *p* = 0.013, η^2^ = 0.098, qualified by an interaction between Group and Version, *F*_(2, 84)_ = 4.639, MSE = 0.194, *p* = 0.012, η^2^ = 0.099. This reflected the fact that patients (*M* = 0.766, *SD* = 0.806) stopped cooking their foods significantly earlier than ideal compared to the controls (*M* = 0.349, *SD* = 0.417) only on the 1-screen version, *t*_(42)_ = 2.156, *p* = 0.039 [2-screen, *t*_(42)_ = 0.690, *p* = 0.494; 6-screen, *t*_(42)_ = −1.048, *p* = 0.301]. While the controls tended to stop the food just as early across the versions, the patients disproportionally stopped the food early on the 1-screen version. The tendency to stop food earlier than ideal decreased sharply with the 2-screen (ABI *M* = 0.597, *SD* = 0.539; Controls *M* = 0.491, *SD* = 0.482) and 6-screen version, so much so that ABI patients stopped food less early than controls on the 6-screen version on average (ABI *M* = 0.228, *SD* = 0.416; Controls *M* = 0.382, *SD* = 0.551).

When people overcooked their foods (i.e., stopped their foods too late), the patients did so significantly more than ideal compared to controls, *F*_(1, 42)_ = 16.598, MSE = 0.571, *p* < 0.001, η^2^ = 0.283 (see Supplementary Table 1 and Figure 4). There was an effect of Breakfast Task Version, *F*_(2, 84)_ = 25.390, MSE = 0.157, *p* < 0.001, η^2^ = 0.377, but no interaction between Group and Version, *F*_(2, 84)_ = 0.435, MSE = 0.157, *p* = 0.649, η^2^ = 0.01. Participants stopped the food later than ideal in the 6-screen (*M* = 1.618, *SD* = 0.44) than the 1-screen (*M* = 1.069, *SD* = 0.692) and 2-screen versions (*M* = 1.129, *SD* = 0.645), *t*_(43)_ ≤ −5.761, *p* < 0.001. The 1-screen and 2-screen did not differ, *t*_(43)_ = −0.762, *p* = 0.45.

#### Average range of stop times

**Figure 5:**
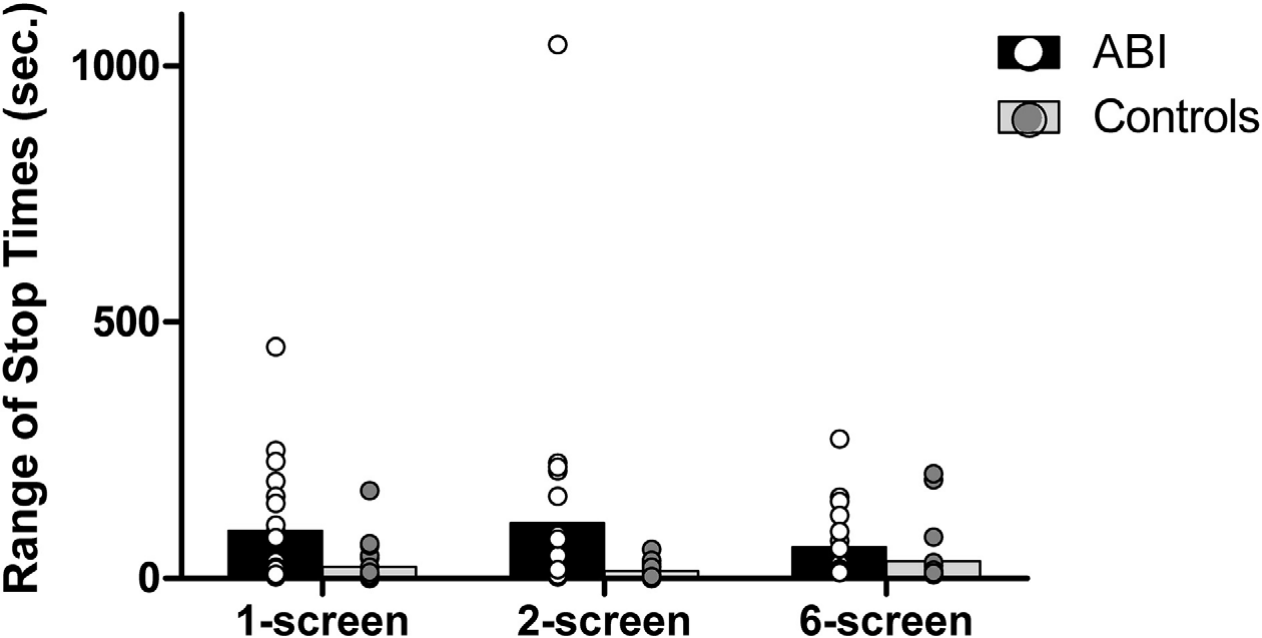
**Range of stop times in seconds**.

The instructions always emphasized the importance of serving the foods at the same time (i.e., of having a range of stop times approaching zero). The aforementioned “discrepancy in cooking time” score and the range of stop times are related but not redundant, in that a person might choose to serve under- or over-cooked foods (i.e., high discrepancy in cooking time), but serve all foods at once (i.e., low average range of stop times). Conversely, a person might choose to serve perfectly-cooked food items (i.e., low discrepancy in cooking time) but not serve all items at the same time (i.e., high average range of stop times). Patients showed a significantly wider range of stop times than controls, *F*_(1, 42)_ = 13.409, MSE = 0.57, *p* = 0.001, η^2^ = 0.242 (see Supplementary Table 1 and Figure 5). The main effect of the Breakfast Task Version showed a trend toward significance, Huynh-Feldt *F*_(1.87, 78.6)_ = 2.576, MSE = 0.159, *p* = 0.086, η^2^ = 0.058 with no interaction, *F*_(1.87, 78.6)_ = 1.359, MSE = 0.159, *p* = 0.262, η^2^ = 0.031.

#### Average deviation of start times

**Figure 6:**
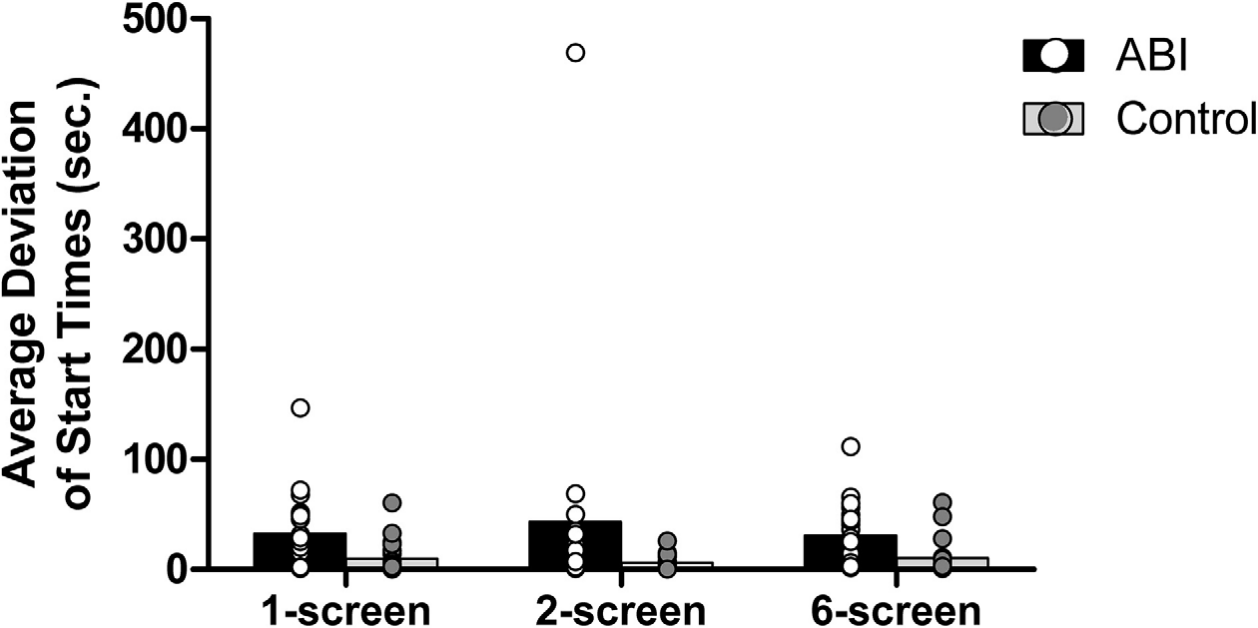
**Average deviation of start times in seconds**.

Each food item has an ideal start time, which is contingent on the previously-started items (except for the eggs, which take 5.5 min to cook and should be started at the onset of the task). The coffee takes 4 min to brew, so the coffee should be started 1.5 min after the eggs. If, for example, the starting time of the coffee is 0.5 min early, what will be the ideal start time of the third food, the sausage (which needs 3.5 min to cook)? In order to reduce the range of stop times, one may decide to start the third food item based on the first item (i.e., 2 min later) or the second item (i.e., 1 min later), or a combination of both (i.e., 1.75 min). The ideal start times for the third, fourth, and fifth food items are an average of the ideal start time based on the first item (e.g., 2 min for the sausage) and the relative ideal start time based on the previous food items (e.g., the actual start time of coffee +0.5 min). Absolute deviations of start time for the food items were then averaged. Patients showed a greater average deviation of start times than controls, *F*_(1, 42)_ = 14.656, MSE = 0.542, *p* < 0.001, η^2^ = 0.259 (see Supplementary Table 1 and Figure 6). No main effect of the Breakfast Task Version, *F*_(2, 84)_ = 1.499, MSE = 0.07, *p* = 0.229, η^2^ = 0.034, and no interaction between Group and Version, *F*_(2, 84)_ = 0.018, MSE = 0.07, *p* = 0.982, η^2^ = 0.000, were found.

#### Early start vs. late start

**Figure 7:**
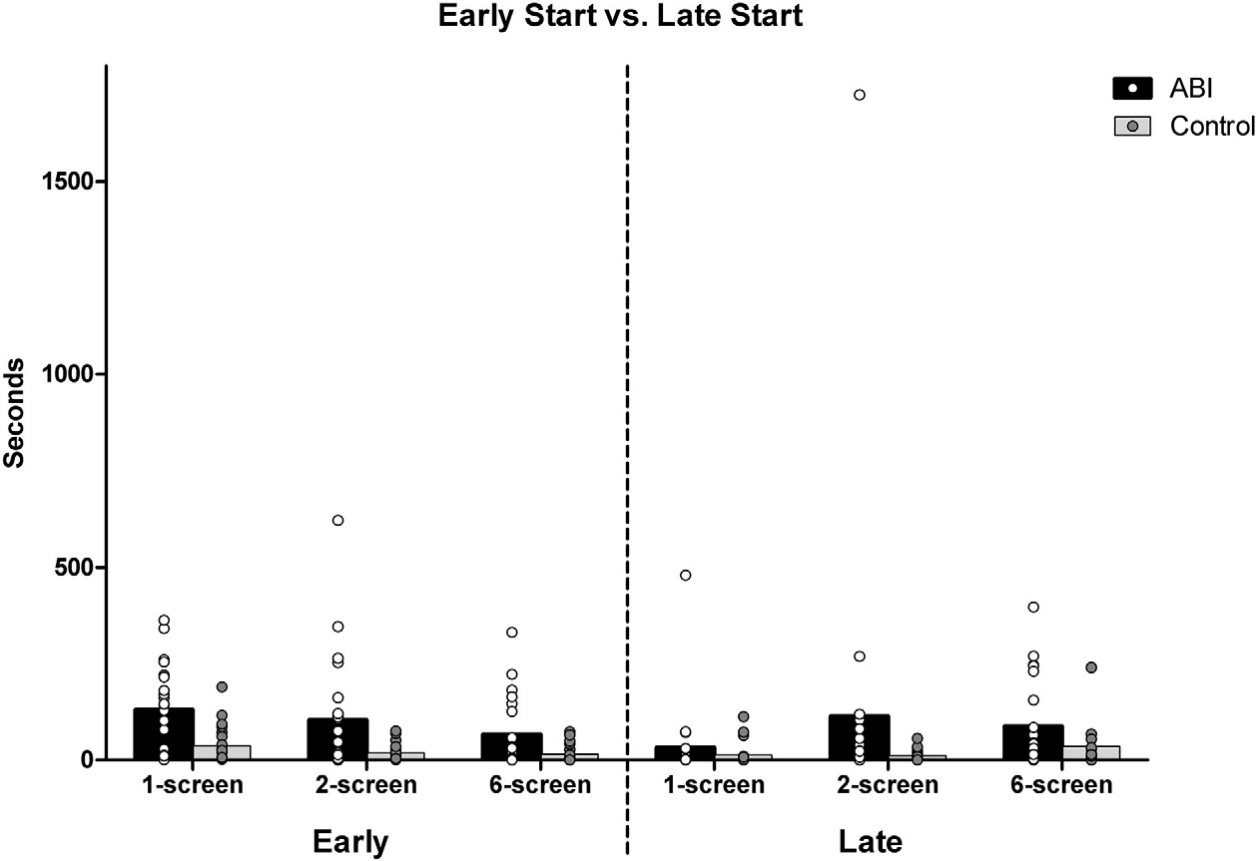
**Average early and late start deviations in seconds**.

The ABI patients missed the ideal start times more than controls, and we further asked whether they started food earlier and/or later than ideal. The food items' ideal start times (as described above) were subtracted from the actual start times. Starting the cooking of a food item early is indicated by an average of the five food items' positive deviations; negative deviations for later than ideal start times. Patients did start the food earlier than ideal compared to the controls, *F*_(1, 42)_ = 7.239, MSE = 1.103, *p* = 0.01, η^2^ = 0.147 (see Supplementary Table 1 and Figure 7). There was an effect of Breakfast Task Version, *F*_(2, 84)_ = 18.252, MSE = 0.175, *p* < 0.001, η^2^ = 0.303, but no interaction with Group, *F*_(2, 84)_ = 0.204, MSE = 0.175, *p* = 0.816, η^2^ = 0.005. The 3 Breakfast Task versions all differed from one another, *t*_(43)_ ≥ 2.742, *p* < 0.009. Participants started foods earlier than their ideal start times on the 1-screen (*M* = 1.524, *SD* = 0.669) than on the 2-screen (*M* = 1.26, *SD* = 0.695) and than on the 6-screen (*M* = 0.985, *SD* = 0.822).

ABI patients started the food no later than ideal compared to controls, *F*_(1, 42)_ = 1.656, *MSE* = 0.834, *p* = 0.205, η^2^ = 0.038 (see Supplementary Table 1 and Figure 7). There was an effect of Breakfast Task Version, Huynh-Feldt *F*_(1.86, 77.95)_ = 11.899, *MSE* = 0.465, *p* < 0.001, η^2^ = 0.221 that did not interact with Group, *F*_(1.86, 77.95)_ = 10.386, *MSE* = 0.465, *p* = 0.666, η^2^ = 0.009. Participants started the foods later than ideal in the 6-screen (*M* = 1.223, *SD* = 0.795) than 2-screen version (*M* = 0.975, *SD* = 0.742), and both later than in the 1-screen version (*M* = 0.547, *SD* = 0.718), *t*_(43)_ ≤ −2.066, *p* ≤ 0.045.

#### Sequencing

Starting or stopping a food a little early or late (while following the proper order of starting eggs, then coffee, sausage, pancakes, and finally toast) could be considered a minor error, but starting foods out of their proper order could be considered a more serious problem. To look at sequencing errors, we subtracted a point on 5 for each food started in incorrect sequence. Participants were not penalized for a previous error, for example if someone began with eggs, then started the sausage, then the coffee (instead of the proper order of eggs, coffee, sausage…), only one point was taken off. All food items other than toast started as the last food item also warranted a penalty. Sequencing errors were relatively rare, especially for controls, and therefore we combined the scores from the 3 Breakfast Task versions and used a Mann-Whitney Test. Patients (Mdn = 14, Minimum = 2, and Maximum = 15, where 15 represents the ideal score) committed more sequencing errors than controls (Mdn = 15, Minimum = 10, and Maximum = 15, where 15 represents the ideal score), *U* = 139.5, *z* = −2.851, *p* = 0.004, *r* = 0.43. Of note, four ABI participants omitted to cook one of the food items in the 6-screen version. None of the controls made such an omission. The closest was a control participant who thought she had pressed the start button for the eggs, but only noticed at the end that she had never actually started them. Rather than deprive her virtual breakfast guests of their eggs, the participant chose to start the eggs and keep setting the table while waiting for them to finish (such a strategy also increased her range of stop times, presented above).

#### Percentage of time spent cooking

**Figure 8:**
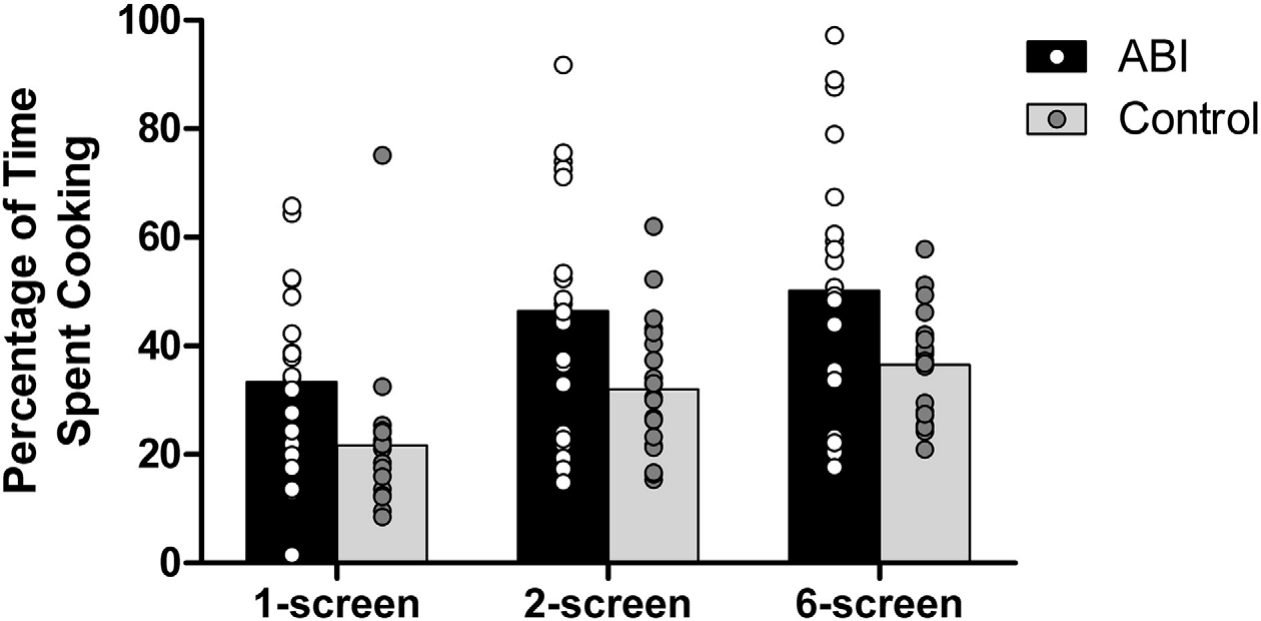
**Percentage of time spent cooking**.

Participants were required to balance their time between cooking and setting as many places at the virtual table as possible. ABI patients spent a greater percentage of their time on the cooking than did the controls, *F*_(1, 42)_ = 7.630, MSE = 570.088, *p* = 0.008, η^2^ = 0.154 (see Supplementary Table 1 and Figure 8; note that because these scores were normally distributed, we computed the ANOVAs on untransformed scores). There was a main effect of Breakfast Task Version, *F*_(1, 42)_ = 5.649, MSE = 67.036, *p* = 0.022, η^2^ = 0.119, with no interaction between Group and Version, *F*_(1, 42)_ = 0.048, MSE = 67.036, *p* = 0.827, η^2^ = 0.001. The 6-screen version (*M* = 43.393, *SE* = 2.703) involved a significantly higher percentage of cooking time than the 2-screen version (*M* = 39.244, *SE* = 2.679). The 1-screen version was not included in the analyses because teasing apart the time spent on table or cooking entails potential inexactitude.

#### Number of table settings

**Figure 9:**
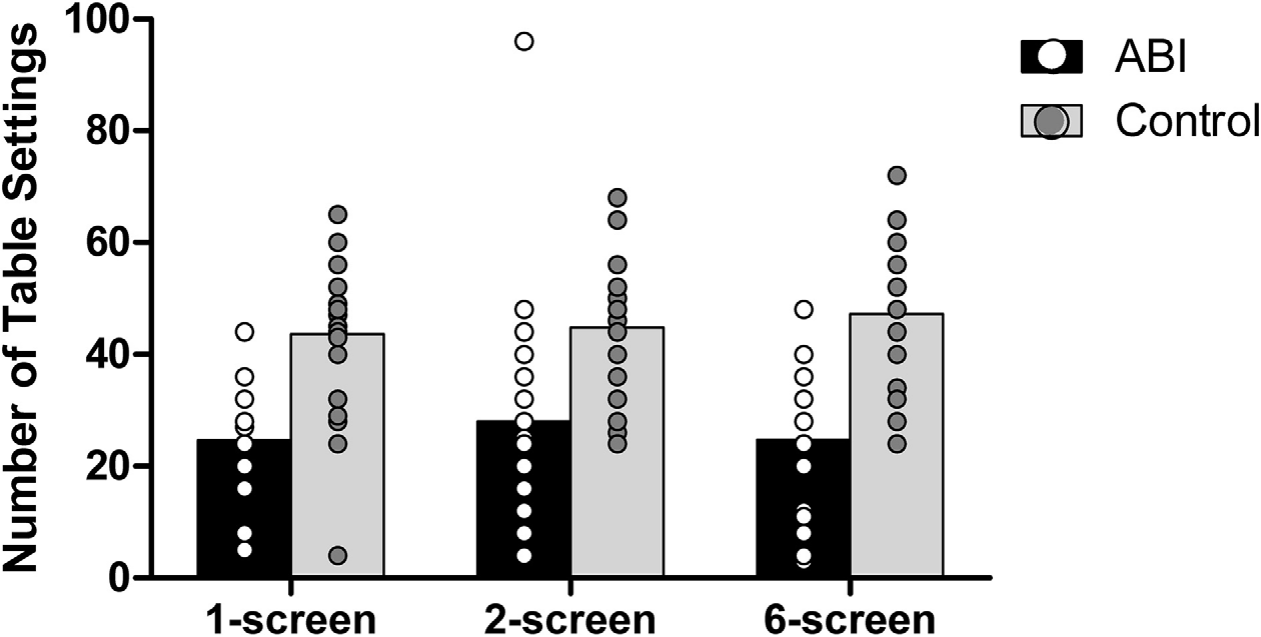
**Number of table settings**.

Patients set fewer places at the virtual table than did controls, *F*_(1, 42)_ = 26.222, MSE = 470.386, *p* < 0.001, η^2^ = 0.384 (see Supplementary Table 1 and Figure 9). There were no main effects of the Breakfast Task Version, *F*_(2, 84)_ = 1.331, MSE = 48.146, *p* = 0.270, η^2^ = 0.031, and no interaction *F*_(2, 84)_ = 1.823, MSE = 48.146, *p* = 0.168, η^2^ = 0.042.

**Figure 10:**
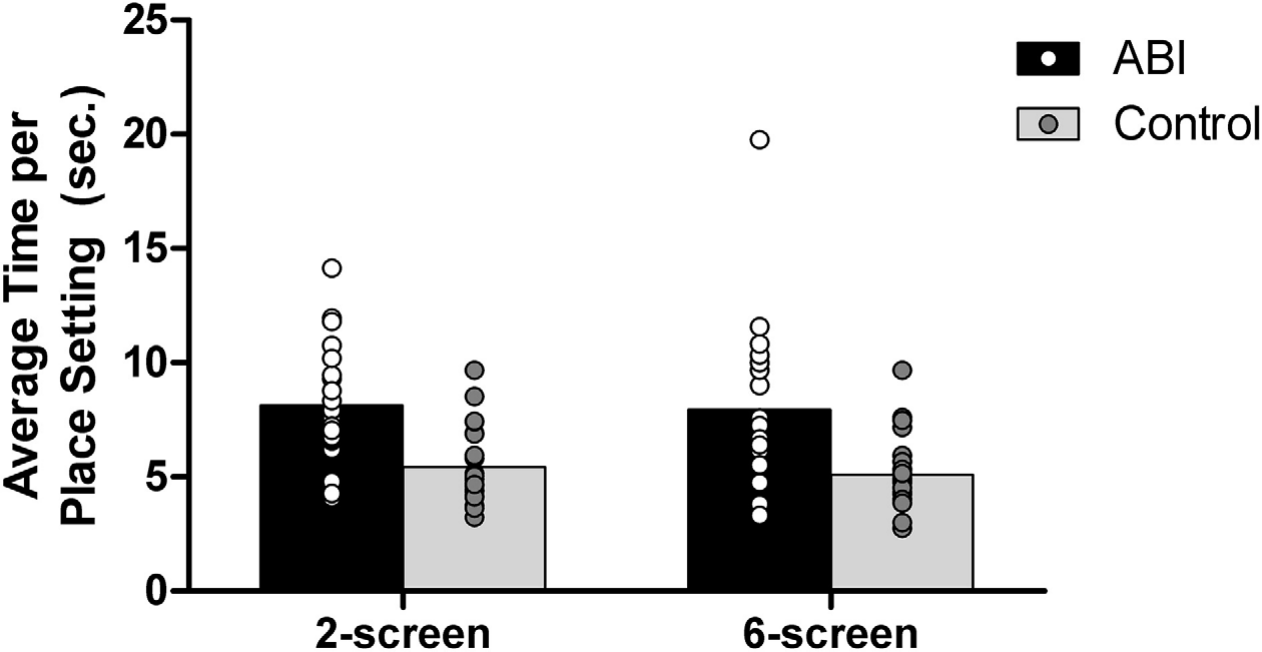
**Average time per place setting in seconds**.

To determine whether patients set fewer table settings relative to the time dedicated to this part of the task, we divided the total time spent on the tables setting by the number of table settings on the 2- and 6-screen versions. The 1-screen version was excluded because of the inherent difficulty in assessing the total time spent on table setting. Patients set fewer places while on the table setting screen compared to controls, *F*_(1, 42)_ = 16.940, MSE = 10.013, *p* < 0.001, η^2^ = 0.287 (see Supplementary Table 1 and Figure 10). The main effect of the Breakfast Task Version was not significant, *F*_(1, 42)_ = 0.667, MSE = 2.181, *p* = 0.419, η^2^ = 0.016, and did not interact with Group, *F*_(1, 42)_ = 0.067, MSE = 2.181, *p* = 0.798, η^2^ = 0.002. The number of table settings and the average time per place setting data fit a normal distribution, so the untransformed data were used in these analyses.

#### Number of food checks

**Figure 11:**
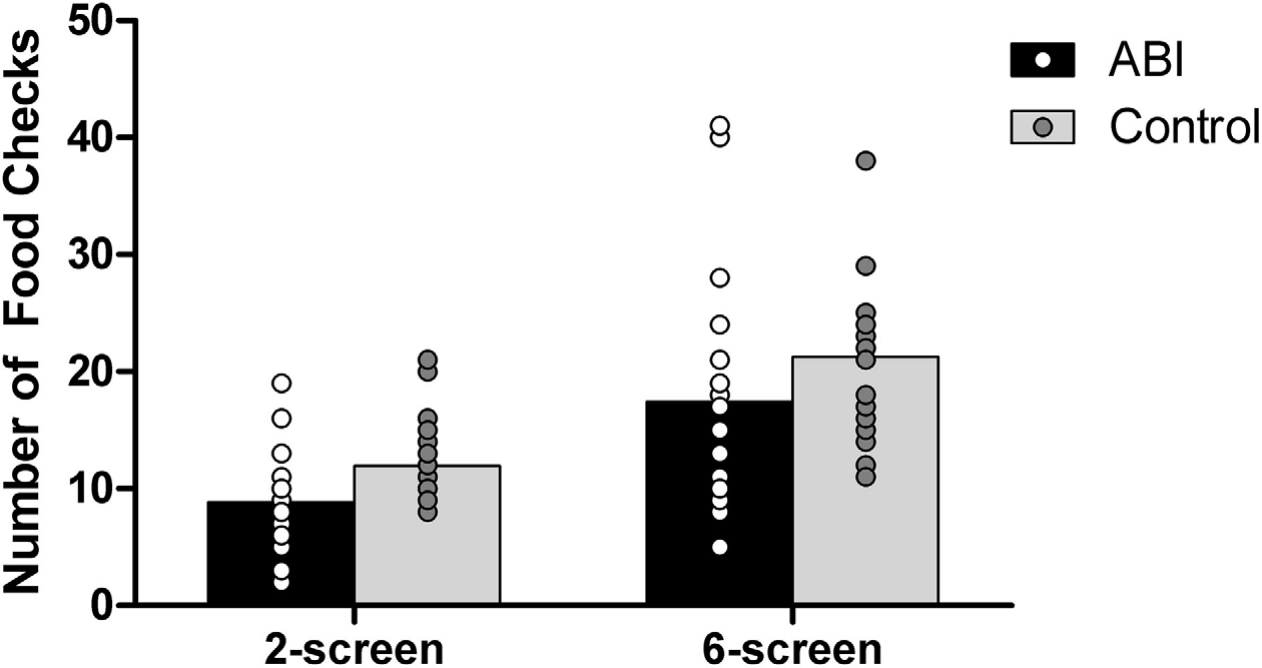
**Number of food checks**.

Controls monitored the cooking of their foods [i.e., switching to the food screen(s) from the place setting screen in the 2-and 6-screen versions] more often than did the ABI patients, *F*_(1, 42)_ = 5.477, MSE = 47.936, *p* = 0.024, η^2^ = 0.115 (see Supplementary Table 1 and Figure 11). Controls and ABI patients made more food checks on the 6-screen version (*M* = 19.364, *SE* = 1.2) than the 2-screen version (*M* = 10.409, *SE* = 0.590), *F*_(1, 42)_ = 57.432, MSE = 30.715, *p* < 0.001, η^2^ = 0.578 [no group interaction, *F*_(1, 42)_ = 0.095, MSE = 30.715, *p* = 0.760, η^2^ = 0.002]. Because food items and the table setting all show on the same screen for the 1-screen version, food checks can be performed by a simple shift of glance and hence cannot be evaluated separately from cooking time within the existing Breakfast Task paradigm.

### Correlations with real world performance

We had expected that ABI patients' performance on the Breakfast Task would be positively correlated with their ability to prepare a real meal. To answer this question, we first had to construct an average overall score:

[0.40(Average discrepancy) + 0.40(Range of stop times) − 0.20(Number of table settings)] + 20

We assigned weights to the three components that made up the overall Breakfast Task score, based on the task instructions, which place greater importance on the cooking (having all foods prepared at the same time, with none over- or under-cooked) than on the table setting. Higher scores for cooking performance (i.e., average discrepancy and range of stop times) reflect poorer performance and higher scores for table setting reflect better performance, so we reversed the table setting score and gave it a lower weight relative to the two other scores. For ease of interpretation, we added 20 to all scores in order to make them all positive (i.e., above zero), and then transformed them to meet assumptions of normality.

To avoid alpha inflation, we summed the scores of the 3 Breakfast Task versions and explored their relationships with the *in vivo* evaluation of independence in cooking and self-report scores. [Separate correlations for each version can be found in Supplementary Table 2.] The self-report measures are composite scales of meal preparation/planning abilities, and a cognitive composite evaluating tasks such as managing finances. Complete data on real world functioning were available for a subset of 16 ABI participants. Patients' actual meal preparation (as assessed by the clinical team) was significantly correlated with their self-reported meal preparation abilities, *rs* = 0.536, *p* = 0.032.

Self-report of meal preparation abilities was significantly correlated with the Overall Breakfast Task score (*rs* = −0.594, *p* = 0.014), along with the aggregate measures of Discrepancy of Cooking Time (*rs* = −0.646, *p* = 0.007), Deviation of Start Times (*rs* = −0.666, *p* = 0.005), Early Start (*rs* = −0.607, *p* = 0.013), and Late Stop (*rs* = −0.760, *p* = 0.001). The patients' independence while preparing real meals (as assessed by the clinical team) was not significantly correlated with their Breakfast Task overall score, *rs* = −0.075, *p* = 0.783.

## Discussion

On the virtual meal-cooking task (the “Breakfast Task,” Craik and Bialystok, 2006), our ABI patients all seemed to have understood the instructions, had the opportunity to go through practice trials before each version of the task, and generally appeared to grasp the gist of what they were to do [which is not surprising, given that semantic knowledge of cooking (including simple script generation) is usually unimpaired in people with brain injuries (Fortin et al., 2003; Godbout et al., 2005; Baguena et al., 2006) and that all of our ABI patients had previous experience with cooking].

Despite this, the patient group showed poorer performance than normal on every Breakfast Task score that we examined. On average, the patients were less likely to have all their foods ready at the same time, over-cooking some foods and under-cooking others. Although some patients were indistinguishable from the healthy participants, up to a third scored outside the range of controls (depending on the particular measure) and we observed some behaviors that truly seemed “dysexecutive.” Moreover, some behaviors suggested not only weaknesses in managing transitions between cooking and table setting (i.e., multi-tasking and prospective memory) but also in forming a valid plan at the outset or keeping such a plan in mind, or both. For example, some patients would run through the practice trials too quickly or begin the task by setting several places at the virtual table rather than by starting the first breakfast item [consistent with Frisch et al. (2012), who found their stroke patients to be less likely to read the instructions carefully before beginning cooking]. Also notable, several of the ABI patients cooked their foods in the wrong sequence, a type of error rarely seen in our controls and not reported at all in the two previous studies with older adults and Parkinson's patients (Craik and Bialystok, 2006; Bialystok et al., 2008). Two patients in particular did not start the eggs first even though these obviously take the longest to cook, and one started all the food items in reverse order from the ideal on one version of the task. Four ABI participants neglected to cook one food item at all on the 6-screen version (three neglecting the last food item, i.e., the toast). Also, occasionally patients would continue setting places at the virtual table after they had stopped cooking all of the food items, or would interrupt their cooking mid-sequence to return to table setting, which was inefficient. Weak performance early in the sequence would bring about a distinct challenge for the patients: Judging the best course of action given that the two primary task objectives (i.e., having the right cooking time for each food, and having all foods finished at about the same time) had now become essentially irreconcilable. Indeed, the mean average deviation in start times for our ABI patients was more than twice as long as that noted for older adults and Parkinson's patients (i.e., 35 vs. approx. 12–15 s; Craik and Bialystok, 2006; Bialystok et al., 2008). Our instructions did not explicitly state what patients should do if they realized later in the task that they had made the error of poor early planning. In future, manipulating instructions and asking patients during or after the task could help elucidate whether they tried to balance the two main objectives of the task, arbitrarily focused on one or the other, or focused specifically on one based on real life priorities. That is, in the last instance, they could have focused on minimizing discrepancies in cooking times over minimizing average range of stop times because of their perception of graver implications of serving under- or over-cooked foods.

Our ABI patients looked quite different from the only other neuropsychological group to have performed the Breakfast task. Bialystok et al. (2008) found that Parkinson's patients performed surprisingly well on the breakfast-making part of the task, but in order to do so they may have strategically ignored the table-setting part of the task. Some of our ABI patients may have been trying to do something similar. For instance, they devoted more time to cooking than to place-setting relative to controls. In fact, on the most challenging version of the task (i.e., the 6-screen version), some ABI patients spent almost all of their time on cooking. Yet, our ABI patients performed significantly more poorly than controls on *both* the cooking and table setting components. The PD patients may have been less impaired than ABI patients overall, or may actually show a different profile of executive impairment than ABI patients (e.g., Zgaljardic et al., 2003). One additional surprising finding in the present study was that although we thought the ABI patients would be most clearly impaired on the 6-screen version of the task (owing to its arguably greater demands on executive functioning), this was not the case. This may stem in part from most of the controls scoring relatively close to zero on several of the Breakfast Task measures (e.g., Discrepancy, Average Deviation of Start and Stop Times), and should be explored further in future.

### The breakfast task reflects multiple aspects of executive functioning

In this study we did not have consistent neuropsychological data available on the patients (owing to significant variability in the time between date of injury/illness onset and neuropsychological testing, as well as variability in the test batteries employed depending on specific diagnoses and by which clinical service they were seen). In future, it would be interesting to know the extent to which traditional executive measures predict Breakfast Task performance. We would note, however, that the Breakfast Task comes from a modern impetus to create relatively realistic, complex tasks that rely on multiple executive functions (e.g., Shallice and Burgess, 1991; Knight et al., 2002; Manly et al., 2002; Lamberts et al., 2010; for a review see Poulin et al., 2013). Such an approach is a double-edged sword: Although it provides participants with an engaging experience with a relatively representative scenario (Burgess et al., 2006), it is not designed to precisely differentiate among the executive processes that might contribute to performance. A happy medium might be to use complex, realistic scenarios such as the Breakfast Task to generate hypotheses about those executive functions that might be impaired in a particular patient, and then isolate those functions using simpler, more traditional measures. Although this approach is potentially useful, it must be borne in mind that these complex scenarios were developed because it might only be on them [i.e., open-ended complex tasks in which a basic context and goal(s) have been provided but important sub-goals may emerge as the task unfolds] that executive problems become apparent (for an impressive attempt to strike a balance, see Wilson et al., 1996).

### The breakfast task as a model of performance in the real world

As the *in vivo* cooking task involved preparing multiple foods for dinner (requiring planning and multi-tasking), the Breakfast Task seemed well-matched to it. We found a significant relationship between ABI patients' self-rated ability to plan and prepare meals and their performance on the Breakfast Task. Because the self-report ratings were taken following several months in a residential rehabilitation program with intensive life skills retraining including weekly meal preparations and feedback about their performance, it could be expected that clients would develop some degree of realistic self-appraisal over time. In keeping with this suggestion, the overall independence scores for *in vivo* cooking did relate positively to patients' self-reports of meal preparation abilities. Yet, surprisingly, overall performance on the Breakfast Task was only weakly (and non-significantly) correlated with overall ratings of independence in preparing a group meal.

The surprisingly low correlation between Breakfast Task performance and real meal preparation is vexing, and although we are not alone in finding this (e.g., Semkovska et al., 2004; Baum et al., 2008; Chevignard et al., 2008, 2010; Yantz et al., 2010; Provencher et al., 2012) we can only speculate as to why it occurred. First, there may actually exist a subtle relationship between these two variables but our subgroup of *n* = 16 patients provided insufficient statistical power to detect it. And yet, other correlations (for example, between patients' self-ratings of meal preparation and their actual performance) were readily apparent even with our small group size. Second, unlike other cooking paradigms (e.g., Neistadt, 1994; Giovannetti et al., 2008), the Breakfast Task primarily measures the executive aspects of cooking, rather than the actual procedures one runs through in the preparation of food (e.g., pouring real coffee, dicing real mushrooms). In this respect, it seemed reasonably well-suited to our ABI patients, who mostly appear to have difficulty with the planning and executive aspects of cooking rather than the procedural ones: Interventions to train our clients on basic tasks such as preparing coffee or a sandwich are not typical, whereas training of complex meal preparation is more commonly needed. Nevertheless, a subset of our ABI patients may have exhibited challenges with the routine procedures involved in cooking (e.g., slowness dicing foods) that were not adequately measured by the Breakfast Task. A more fine-grained assessment of their *in vivo* cooking might help us distinguish patients who appear to have more “executive” problems when cooking from those who have more “procedural” problems, with patients in the former group perhaps showing a closer correspondence between real world and Breakfast Task performance. Third, our ratings of *in vivo* cooking were relatively coarse, using 4-point scales limited to two specific dimensions (i.e., speed of execution, and use of compensatory strategies) and a global one, in keeping with the fact that ratings were made on the basis of summary narratives drawn from reports. Despite the advantage of our summary narratives being based on multiple samplings of meal preparation, the scale that we used here included less detailed information than other coding methods (e.g., Giovannetti et al., 2008; Frisch et al., 2012). Finally, in some ways, the *in vivo* task and computerized tasks were different from one another. Notably, the *in vivo* assessment placed considerable value on the use of compensatory strategies to mitigate risk and produce a good meal, with no direct parallel on the Breakfast Task. One solution might be to separately assess procedural aspects of cooking using a basic task, as a baseline from which to gauge the role of “executive” problems in a more complex one (see Schmitter-Edgecombe et al., 2011 for a good example of such tasks).

Despite this surprising null correlation, to our minds the Breakfast task shows potential and deserves further work. All of the ABI patients we recruited could understand and adequately perform the task, almost all (22 out of 25 = 88%) completed it, and many found it to be enjoyable and engaging. In general, computerized assessment of complex tasks such as meal preparation has real advantages over *in vivo* assessment, including standardized administration and scoring, reduced potential for scorer bias, the collection of more data than one observer could possibly generate (even with video-recording for later analysis), millisecond timing (which is impossible with scoring real or video-recorded sessions), indirect observation (instead of needing to be quite close to the food and the client for *in vivo* analysis), the relative independence of administration (e.g., a single tester is required to explain and start the task and then can keep an eye on performance while carrying out other duties), portability (i.e., the assessment does not require a real kitchen), and the possibility of varying task demands easily and uniformly across participants (e.g., using 3 versions, as in the present study). Although video recordings may be useful for less fine-grained analyses or for establishing the merits of a scoring system, the regular need for video recordings to apply such a method would typically be prohibitive within many clinical settings for the aforementioned reasons.

## Future work

Notwithstanding the potential advantages of computerized assessment, more work is required before replacing traditional *in vivo* evaluation methods. In particular, one strategy to learn more about the correspondence between computerized and real-world cooking might be to make the computerized and real world tasks more similar to one another: How would the patients perform if asked to cook a real breakfast of eggs, coffee, sausage, pancakes, and toast in 5.5 min in the kitchen? One could also use virtual reality to make the computerized task look and feel as similar as possible to real life. Several reports have recently emerged of strong correlations between real and virtual performance of executively-demanding scenarios, including cooking (e.g., Zhang et al., 2003; Renison et al., 2012). Virtual reality assessments retain most if not all of the advantages of other kinds of computerized assessment, and will undoubtedly become less expensive and easier to administer in the near future.

By comparing the breakfast simulation task with *in vivo* cooking, the present study has helped us identify important translational issues. Use of simulation tasks requires careful consideration of how patients, under certain circumstances, may intentionally deviate from instructions aimed at weighted sampling of various cognitive abilities, drawing instead on affordable knowledge about *cooking for people*. One important question concerns to what extent the task and its instructions should constrain or anticipate and allow changes in approach to the task as it is unfolding based on principles of real world cooking and human need. For example, the importance of being able to detect mid-task adjustments vs. poor or random performance seems sensible not only in light of the application of common sense notions and options available to us in real life cooking but also when one considers the arithmetical and working memory burden associated with resetting “ideal” start times of later food items via the expectation of an elaborate mathematical averaging process should a person err with food sequences or start times early in the task. Placing considerable effort on estimating new ideal start times for later foods based on error introduced with early food start times would not necessarily constitute the best “real life” strategy because of the information processing load, proving detrimental to the allocation of cognitive resources to other ongoing aspects of the task and only aggravating matters. Future studies should measure process-related (i.e., interpretative) issues, likely through questioning of patients both during training as well as following testing. Finally, as the focus of such studies shifts toward predicting real life performances, there may be a need for a parallel shift in focus from measuring cognitive impairment to one of measuring disability. Building on clients' abilities to adapt their approach to tasks using compensatory strategies is a cornerstone principle in the rehabilitation of neurologically compromised individuals. In future research we will need to consider how to incorporate such opportunities into simulation tasks as well.

## Author contributions

Annick N. Tanguay tested participants, ran statistical analyses, and contributed to writing the manuscript. Patrick S. R. Davidson co-conceived the project and contributed to writing the manuscript. Karla V. Guerrero Nuñez tested participants and contributed to writing the manuscript. Mark B. Ferland co-conceived the project and contributed to writing the manuscript.

### Conflict of interest statement

The authors declare that the research was conducted in the absence of any commercial or financial relationships that could be construed as a potential conflict of interest.
