# Supplementary material for: Cooking breakfast after a brain injury
Source: Front Behav Neurosci. 2014 Sep 2;8:272. doi: 10.3389/fnbeh.2014.00272 (PMC4151095; doi:10.3389/fnbeh.2014.00272)
Supplement: Supplementary file 1 [file DataSheet1.ZIP › 93283_Davidson_Table_S1.PDF]

Appendix

Table 1. Group Effects on the Breakfast Task.

Breakfast Task Measures	Description		F	MSE	P	η^2
Total Task Time	Time from first to the last action.	Closest to 5.5 minutes is best.	2.161	.009	.149	.049
Average Discrepancy	The average discrepancies between the actual and ideal cooking times of each food item.	Lower is better.	21.403	.295	.001	.338
Early Stopping	Negative discrepancies from the ideal cook times.	Lower is better.	.956	.523	.334	.022
Late Stopping	Positive discrepancies from ideal cook times.	Lower is better.	16.598	.571	.001	.283
Average Range of Stop Times	The difference between the times of the first food stopped and the last one.	Lower is better.	13.409	.570	.001	.242
Average Deviation of Start Times	The average differences between the actual and ideal start times of each food item.	Lower is better.	14.656	.542	.001	.259
Early Start	Negative deviations from the ideal start times.	Lower is better.	7.239	1.103	.01	.147
Late Start	Positive deviations from the ideal start times.	Lower is better.	1.656	.834	.205	.038
Percentage of Time Spent Cooking	Percentage of time spent on cooking.	It depends.	7.630	570.008	.008	.154
Number of Table Settings	Number of utensils/plates set.	Higher is better.	26.222	470.386	.001	.384
Average Time per Place Setting (sec)	Total time spent on table setting divided by the number of table settings.	Lower is better.	16.940	10.013	.001	.287
Average Food checks	Number of alternations from the table setting screen to the cooking screen(s).	It depends.	5.477	47.936	.024	.115
