# Supplementary material for: Cooking breakfast after a brain injury
Source: Front Behav Neurosci. 2014 Sep 2;8:272. doi: 10.3389/fnbeh.2014.00272 (PMC4151095; doi:10.3389/fnbeh.2014.00272)
Supplement: Supplementary file 1 [file DataSheet1.ZIP › 93283_Davidson_Table_S2.pdf]

Table S2. Correlation matrix for each of the Screen versions of the Breakfast Task

	Overall Score	Average Discrepancy	Range of Stop	Deviation of Start Times	Number of Table Settings	Percentage of Cooking Time	Early Stop	Late Stop	Early Start	Late Start	Total Task Time	Time per table setting
**Breakfast Task – 1-screen Version**												
In-vivo evaluation	-.112	.406	.011	-.133	.288	-.414	.151	.083	-.042	-.458	-.340	-.431
RADLS Meal Prep/Plan	-.498	-.253	-.303	-.676**	-.122	.258	-.045	-.340	-.618*	-.333	-.379	-.185
**Breakfast Task – 2-screen Version**												
	Overall Score	Average Discrepancy	Range of Stop	Deviation of Start Times	Number of Table Settings	Percentage of Cooking Time	Early Stop	Late Stop	Early Start	Late Start	Total Task Time	Time per table setting
In-vivo evaluation	-.226	-.084	-.031	-.120	.340	-.283	-.053	-.045	-.279	.137	.355	-.046
RADLS Meal Prep/Plan	-.657**	-.604*	-.460	-.716**	-.158	.347	-.489	-.561*	-.548*	-.410	-.087	.042
**Breakfast Task – 6-screen Version**												
	Overall Score	Average Discrepancy	Range of Stop	Deviation of Start Times	Number of Table Settings	Percentage of Cooking Time	Early Stop	Late Stop	Early Start	Late Start	Total Task Time	Time per table setting
In-vivo evaluation	-.211	-.214	-.117	-.279	.223	-.137	-.139	-.341	-.403	.256	.125	-.165
RADLS Meal Prep/Plan	-.744**	-.613*	-.485	-.711**	-.309	.463	-.277	-.598*	-.514*	-.153	-.289	.087

N=15; *p<.05, **p<.01
